# Automated Multi-Peak Tracking Kymography (AMTraK): A Tool to Quantify Sub-Cellular Dynamics with Sub-Pixel Accuracy

**DOI:** 10.1371/journal.pone.0167620

**Published:** 2016-12-19

**Authors:** Anushree R. Chaphalkar, Kunalika Jain, Manasi S. Gangan, Chaitanya A. Athale

**Affiliations:** Div. of Biology, IISER Pune, Pashan, Pune, India; Beijing Forestry University, CHINA

## Abstract

Kymographs or space-time plots are widely used in cell biology to reduce the dimensions of a time-series in microscopy for both qualitative and quantitative insight into spatio-temporal dynamics. While multiple tools for image kymography have been described before, quantification remains largely manual. Here, we describe a novel software tool for automated multi-peak tracking kymography (AMTraK), which uses peak information and distance minimization to track and automatically quantify kymographs, integrated in a GUI. The program takes fluorescence time-series data as an input and tracks contours in the kymographs based on intensity and gradient peaks. By integrating a branch-point detection method, it can be used to identify merging and splitting events of tracks, important in separation and coalescence events. In tests with synthetic images, we demonstrate sub-pixel positional accuracy of the program. We test the program by quantifying sub-cellular dynamics in rod-shaped bacteria, microtubule (MT) transport and vesicle dynamics. A time-series of *E*. *coli* cell division with labeled nucleoid DNA is used to identify the time-point and rate at which the nucleoid segregates. The mean velocity of microtubule (MT) gliding motility due to a recombinant kinesin motor is estimated as 0.5 μm/s, in agreement with published values, and comparable to estimates using software for nanometer precision filament-tracking. We proceed to employ AMTraK to analyze previously published time-series microscopy data where kymographs had been manually quantified: clathrin polymerization kinetics during vesicle formation and anterograde and retrograde transport in axons. AMTraK analysis not only reproduces the reported parameters, it also provides an objective and automated method for reproducible analysis of kymographs from *in vitro* and *in vivo* fluorescence microscopy time-series of sub-cellular dynamics.

## Introduction

Kymographs, or space-time plots, have been extensively used to analyse sub-cellular microscopy time-lapse data with improvements in microscopy. It has been used in the past to characterize organelle transport, cell division and molecular motor motility as reviewed by Pereira et al. [1], and the wide-range of applications could be the result of the reduced spatial dimensions of complex microscopy time-series. Most often however, kymography has been used as a qualitative readout of movement or dynamics. In studies where kymographs have been quantified, most often this has been manual, as seen in the Multi Kymograph plugin for ImageJ [2]. Most of the existing tools such as the automated kymography tool [3] and ‘guided’ kymography [1] focus on automating the process of kymograph building. Few methods for the automated quantification of kymographs exist, such as ‘Kymomaker’ [4] and a curvelets based tool [5]. Both these tools automate quantification, but cannot deal with merging and spitting events. Despite the ubiquitous nature of merging and splitting events in typical sub-cellular processes, none of the existing tools for the automated quantification of kymographs include a feature to handle budding and coalescence.

Genome segregation is conserved across cellular systems and has been extremely well studied in the rod-shaped Gram-negative bacterium *Escherichia coli* [6,7]. However microscopic analysis of DNA segregation has only recently been made possible with improvements in microscopy and image-analysis [8–10]. Given the almost 1D geometry of segregation of the genome along the long axis of the cell, kymography is a convenient way to analyze the process of nucleoid DNA segregation. Recent studies using explicit 3D over time tracking have found compaction waves are associated with *E*. *coli* genome segregation [11]. Based on a reduction in dimensions to 1D over time, a quantitative kymograph-based analysis could be used to screen for changes and defects in segregation, without the need for more complex datasets and their analysis.

The process of microtubule transport by molecular motors reconstituted *in vitro*, referred to as a `gliding assay’ has been extensively used to examine the fundamental nature of multi-molecular transport of actin and microtubule filaments by motors [12–15]. Recent studies have also used ‘gliding assays’ to address microtubule mechanics based on the bending of filaments while undergoing transport [16]. Kymography of cytoskeletal filaments *in vivo* has been used to follow actin contractility and microtubule buckling dynamics [17]. However in most cases the use of kymography has been limited to visualizing the time-series in a single-image, as a compact form of data representation. A general tool that could use this information to objectively extract the measures of motility would hence be of some use to these multiple applications.

The assembly of proteins by ‘recruitment’ to structures is fundamental in multi-protein complex formation. The assembly of vesicles by budding off membranes and their fusion is critical for cellular function. For the assembly of coated pits with clathrin for endocytosis the site of assembly [18], sequence of binding events [19] and interactions of other proteins [20] is considered to be critical. Microscopy of *in vitro* reconstituted membrane bilayers has become a powerful tool to study the dynamics of protein assembly during vesicle formation [21,22]. Proteins such as epsin, which were reported to accelerate clathrin ‘recruitment’ [23] have been examined using kymography of the fluorescently labelled clathrin and the effect of mutant epsins on the process [24]. While such an approach lends itself to high-content screening, the analysis of the kymograph has been manual. Many other such ‘recruiment’ dynamics studies could benefit from an automated routine to quantify the kinetics of assembly through intensity measurements coupled to kymography.

Neuronal vesicles are transported in axons by the action of molecular motors. Microscopy of *in vitro* reconstituted [25] and the *in vivo* transport in cultured cells [26,27] has provided insights into both the components and forces regulating transport. Recent technical developments have allowed whole animal *in vivo* microscopy of sub-cellular vesicle movements in neurons [28]. In this and comparable studies, quantitative statistics have been obtained using manual detection of kymographs. This is possibly due to the complex nature of the time-series with cross-overs and the crowded *in vivo* environment. An approach that uses objective criteria and automates the process of quantification could provide valuable improvements to our understanding of fundamental nature of vesicle transport as well as aid in the process of modeling vesicle transport.

Here, we have developed a novel tool to automatically quantify kymographs from fluorescence image time-series. We proceed to demonstrate the utility of the automated multi-peak tracking kymography (AMTraK) tool by quantifying dynamics from diverse sub-cellular fluorescence microscopy data sets. These include bacterial genome-segregation, microtubule (MT) motility of 1D filaments and 2D radial asters, membrane protein assembly dynamics and vesicle transport in axons.

### Algorithm and workflow

The automated multi-peak tracking kymography (AMTraK) is open source software based on an algorithm that combines peak detection and distance minimization based linking to quantify dynamics of fluorescence image time-series. The source code has been released with a GPL license and can be accessed from: http://www.iiserpune.ac.in/~cathale/SupplementaryMaterial/Amtrak.html and https://github.com/athale/AMTraK

**Fig 1:**
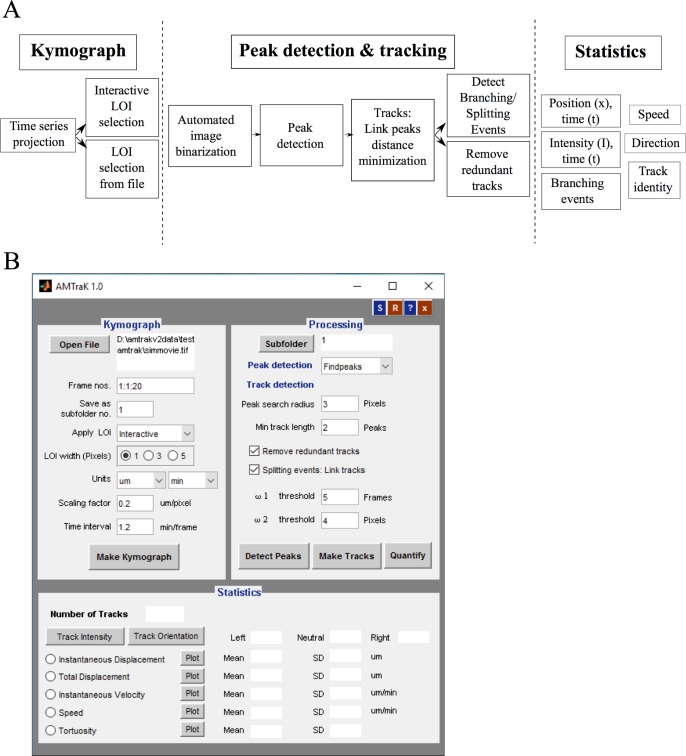
Algorithm workflow and user-interface. (A) The workflow of the algorithm involves three steps (1) kymograph generation, (2) peak detection and tracking and (3) quantification and the functions invoked by each part are elaborated. (B) The GUI is organized to reflect this workflow.

The program has a GUI front-end and is accompanied by a detailed help file. The algorithmic workflow (Fig 1A) is divided broadly into three steps:

1. Making the kymograph
2. Peak detection and tracking
3. Statistics

These steps in the workflow are reflected in the graphical user interface (GUI) layout (Fig 1B). The functioning of each of these steps is briefly described as follows:

#### (a) Making the kymograph

The user chooses an input image time-series with the “Open File” button. Image time-series are assumed to be uncompressed, multi-page TIF files (independent of bit depth). The user can choose to process either the whole or a subset of frames using the “Frame nos.” text box. For example entering “2:2:8” will now result in only frames 2, 4, 6 and 8 being processed for further analysis. The text box “Save as sub-folder” takes a number input (default “1”) indicating where the outputs will be stored (e.g.: “./amtrak-1”). The drop-down menu “Apply LOI” allows the user to either choose a line of interest (LOI) using the mouse (“Interactive”) or apply a pre-existing LOI on a different channel (color) of the image time-series (“From file”). Once an interactively drawn LOI is selected, it is stored in the output sub-folder as “LOIselection.txt” (S1 Data). This LOI can subsequently be applied, to another channel or the same region of another dataset (e.g.: microfluidics channels) using the “From file” mode. For this, the user is required to load a separate TIF time-series using “Open File” and change the sub-folder number in order to prevent overwriting old data. The “LOI width (pixels)” allows a user to choose the width of the LOI, to compensate for occasional drift of the object, in a direction orthogonal to the LOI orientation. The choice widths- 1, 3 and 5 pixels- is centered around the selected LOI pixels, similar to that implemented in the ImageJ Multi Kymograph plugin [2]. The drop-down menu “Units” allows the user to select distance and time units, and the text boxes “Scaling factor” and “Time interval” are used to provide conversion factors per pixel and frame respectively. This results in scaling the pixels and frame numbers to physical units. The button “Make Kymograph” produces a maximum intensity projection image of the input time-series, if the user had chosen the “Interactive” mode (default) in the “Apply LOI” menu. The user is required to select the line of interest by drag-clicking the mouse. Double-clicking ends the selection, and throws a dialog box, which prompts the user to choose to either select more LOIs or continue with the processing of the one already selected. This generates file one or more “LOIselection.txt” files in the sub-folders. If the “From file” mode was selected, the program allows the user to select a pre-existing “LOIselection.txt” from the directory structure. The program then generates kymographs based on these LOIs and stores the matrices corresponding to the LOIs in sub-folders numbered according to the sequence of LOI selection (e.g.: “/amtrak-1/”, “/amtrak-2/” etc.).

#### (b) Peak detection and tracking

*Detecting peaks*: The button “Subfolder” allows the user to choose the kymographs to be processed using “Add”, which adds the subfolders created earlier to the active list. Using this feature, a user can either process a single kymograph at a time, or process multiple kymographs using the same parameters. The kymograph is segmented row-wise using Otsu’s method [29] and the resulting binary image is processed for “Peak detection”. The user can choose between three alternative methods: (i) *findpeaks* [30] and (ii) watershed [31] to find central peaks, while (iii) Canny edge detection [32] is useful if the edge information is the most reliable descriptor of the dynamics. Typically *findpeaks* and watershed are ideal for spherical objects.

*Linking*: The list of peaks *P(t)* for each time point *t* is linked resulting in tracks, based on user input parameters of “Peak search radius” (λ_1_) and “Min. track length” (λ_2_). Peaks are linked if the minimal pair-wise distance dj(t,t+τ) between every *j*^th^ peak in successive rows (*t*, *t+*τ) satisfies the condition min(d_j_(t, t+τ)) ≤ λ_1_, iteratively for the *j*^th^ peak in every subsequent time step (t+τ). If two or more peaks are equidistant, the peak that makes the largest angle (0 to π) with the existing track is chosen, similar to our previously developed branch detection method [33]. For the peaks in t = 1, the angle criterion does not hold true and equidistant peaks are resolved by user-input. Tracks are eliminated from further analysis if their number of peaks linked len(P) ≤ λ_2_, to avoid artifacts due to very short tracks.

*Remove redundant*: If the checkbox “Remove redundant tracks” is selected, each i^th^ track with η_i_ coordinates, is tested for intersections using the inbuilt *intersect* function. If the number of common coordinates η_c_ satisfies the condition η_c_ ≥ η_i_/3, it is eliminated as a redundant track.

*Splitting and joining tracks*: If the checkbox “Splitting events: Link tracks” is selected, events where two tracks merge are identified by a two-step process. First, all peaks (I(x,t)) are evaluated for the condition I(x,t) = (d^t^e ≤ ω_1_) AND (d^x^e ≤ ω_2_), where d^t^e is the distance on the time-axis (t) and d^x^e is the distance on the spatial (x) axis. Then, a peak with the minimal (Euclidean) distance is minimized for the distance to the end-point (e) coordinate J_m_. The time and distance thresholds are set by the user in the text box for ω_1_ (frames) and ω_2_ (pixels) respectively.

The button “Detect Peaks” then outputs an image of the kymograph with the peaks overlaid in color, while invoking the button “Make tracks” links the peaks based on the input parameters. Lastly the button “Quantify” produces a text file corresponding to each track (S2 Data, S3 Data and S4 Data).

#### (c) Statistics

This section of the code produces both text-file outputs and plots of the dynamics estimated from the kymograph. The frequency distribution of “Instantaneous Displacement”, “Total Displacement”, “Instantaneous Velocity”, “Speed” and “Tortuosity” (i.e. directionality) are plotted if the button “Plot” corresponding to these variables is pressed. Additionally the mean and standard deviation (s.d.) of these variables are also generated in the text boxes. Pressing the “Track Intensity” button plots the normalized (0–1) grey value intensity of each track as a function of the time. The button “Track orientation” triggers a recoloring the tracks in the kymograph based on the net direction of movement along the X-axis- blue (-ve, left), red (+ve, right) and green (stationary, neutral).

The outputs of the analysis are stored in multiple tab-delimited text files: “LOIselection.txt” with the LOI coordinates (S1 Data), “USER_TrackStats.txt” which reports track-wise mean values (S2 Data), “USER_InstStats.txt” which reports the time-dependent statistics (S3 Data), “Tracklist.txt” which stores the grey-value intensities as a function of track number and time (S4 Data) and “Branchpoints.txt” which stores the position and time coordinates of detected branches (S5 Data). The user-inputs to AMTraK in terms of files, directories and parameters are all stored in “All_Parameters.txt” (S6 Data), to enable reproducible analysis.

## Materials and Methods

### Simulated test images

Simulated images of static beads were generated by creating 8 bit images with a black background (intensity: 0) with equally spaced single white pixels (intensity: 255) in MATLAB (MathWorks Inc., USA). To simulate bead motion, a simple 1D random-walk was implemented where each bead was moved randomly in each frame, with displacement drawn from a normally distributed random number with mean m = 0 and standard deviation (s). The standard deviation is a measure of the mean speed of motion. Both the static and mobile bead image time-series were filtered with a 5x5 disk filter and smoothed using a 3x3 averaging filter. The resulting convolved circular objects (S1A Fig) have intensity profiles that resemble point sources of fluorescence signal (S1B Fig). The time-series were saved as a multi-page TIF files. Noise was added to individual time-series in order to simulate increasing levels of image-noise using a Gaussian filter with increasing standard deviation (0–100) using ImageJ [34].

### Bacterial growth and microscopy

*E*. *coli* MG1655 (CGSC, Yale, USA) expressing the pBAD24-hupA-GFP [35] were cultured in Luria Bertani (LB) medium (HiMedia, Mumbai, India) with 100 μg/ml Ampicillin (Sigma-Aldrich, Mumbai, India) at 37°C with shaking at 170 rpm (Forma, ThermoScientific, USA). Nutrient ‘agar-pads’ with 0.2% arabinose (Sisco Research Labs, Mumbai, India) and 100 μg/ml ampicillin were imaged on a glass-bottomed Petri dish (Corning, NY, USA) at 37°C using an inverted Zeiss LSM780 confocal microscope (Carl Zeiss, Germany) with a Plan Apochromat 63x (N.A. 1.40, oil) lens in DIC and fluorescence (excitation by 405 nm diode laser with a beam splitter MBS 405 and the emission collected between 487–582 nm) modes. Images were corrected for drift using the rigid body transformation in the StackReg plugin [36] for ImageJ.

### Microtubule gliding assay

A 1:4 ratio of TRITC-labeled bovine and unlabeled porcine tubulin (Cytoskeleton Inc., USA) at a concentration of 20 μM were used to prepare taxol stabilized MT-filaments in general tubulin buffer as described by the supplier (Cytoskeleton Inc., USA). Into a double backed tape chamber, we sequentially flowed in 4.1 μg/μl of a 67 kDa recombinant human kinesin (Cytoskeleton Inc., USA), blocking buffer (5 mg/ml Casein) and MT filaments. The chamber was then washed with a casein-containing buffer and the reaction was started with 1 mM ATP with anti-fade mix (0.05 M glucose, 1% sucrose, 0.5 mg/ml catalase, 0.5 mg/ml glucose oxidase, 0.5% beta-mercaptoethanol (Cytoskeleton Inc., USA)). Time-series images were acquired every minute for 30 minutes on an upright epifluorescence microscope with a 40x (N.A. 0.75) EC Plan Neofluar lens mounted on a Zeiss Axio Imager.Z1 (Carl Zeiss, Germany) using filters for excitation (563 nm) and emission (581 nm) and an MRC camera (Carl Zeiss, Germany).

### Image processing

The acquired time-series and movies taken from published data were converted to uncompressed TIF time-series using ImageJ (Schneider et al., 2012) and online converters for MOV files. MT-gliding assay images were de-noised using a median filter in ImageJ. For manual analysis of kymographs of MT-gliding, a program was written in MATLAB (MathWorks Inc., USA) to generate a kymograph from the time-series, interactively draw a segmented line along the edges and extract coordinates to calculate velocities. The automated multi-peak tracking kymography (AMTraK) code was implemented in MATLAB R2014b (MathWorks Inc., USA) in combination with the Image Processing (ver. 7.0) and Statistics (ver. 7.3) Toolboxes and tested on Linux, Mac OSX and Windows7 platforms. Vesicle transport image time-series in *C*. *elegans* from supporting material of published work [28] were calibrated based on the width of the axon from the same report.

### Data analysis

All data analysis and plotting was performed using MATLAB 2014b (MathWorks Inc., USA). Fitting of custom functions was performed using either the Levenberg-Marquardt non-linear least square routine or the Trust-Region method, implemented in the CurveFitting toolbox (ver. 3.5) of MATLAB.

## Results

### Accuracy of detection

**Fig 2:**
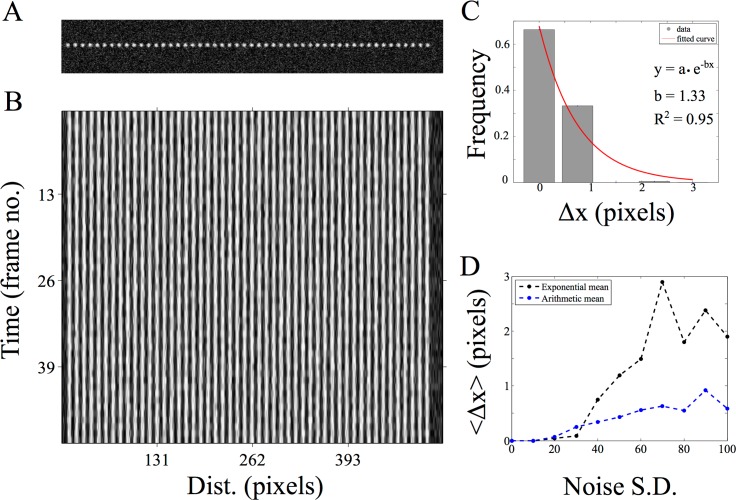
Estimating positional accuracy. (A) A single frame of a 2D image time-series of static spheres (with a peak intensity of 1) with Gaussian noise (mean = 0, s.d. = 40) is analyzed using AMTraK (B) resulting in a kymograph. (C) The frequency distribution of the error in position detection (Δx) by AMTraK (bars) is fit by an exponential decay (red). The mean error obtained is 0.75 pixels (goodness of fit R^2^ = 0.95) for a representative time-series with noise s.d. = 40. (D) The mean error of detection (y-axis) from the exponential fit <Δx> = 1/b (black) is compared to the arithmetic mean (blue) in pixel units, plotted as a function of increasing noise s.d. (x-axis). The noise generates random intensities drawn from a Gaussian distribution with mean 0 and the specified s.d. being added to the image (based on the “Specified Noise” function in ImageJ).

**Fig 3:**
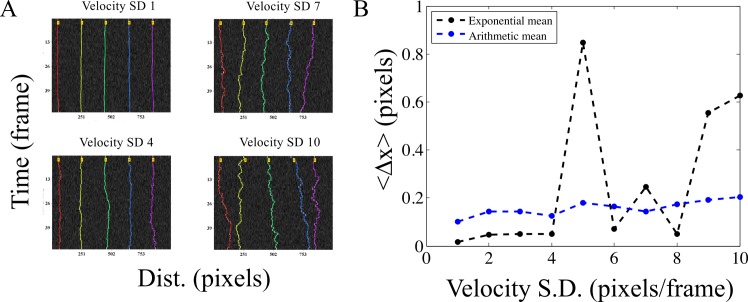
Positional accuracy of tracking simulated motility. (A) Kymographs of time-series of spheres undergoing a 1D random walk with Gaussian noise (s.d. = 30) were tracked. The colors indicate the detected tracks. (B) The arithmetic mean (blue) and exponential mean (black) of error in position detection (Δx) (y-axis) over 3 iterations of the time-series is plotted for increasing velocity of the random-walk (x-axis) as inferred from the standard deviation (s.d.).

To test the positional detection accuracy of the algorithm, we have created simulated image time-series of circular objects that represent typical fluorescence images of circular objects (Fig 2A), comparable to images of sub-cellular structures in pixels (S1A Fig). Since the time-series consists of the same image, the objects are perfectly static as seen in the resulting kymograph (Fig 2B) output from running AMTraK on the data. Intensity variations are a result of the noise from the spatial filter (s.d. 40). The difference between the position of the detected tracks (x_D_) and the simulated position (x_S_) is used as an estimate of the limit of accuracy in position detection, Δx = |x_S_-x_D_|. The normalized frequency distribution of Δx can be fit to an exponential decay function to obtain a mean accuracy <Δx> = 1/b from the fit, in pixel units (Fig 2C). For all images with noise of s.d.< 40, the mean error (from fit) in detection 〈Δ*x*〉<1 pixel. For higher values appears to saturate between 2–3 pixels (Fig 2D). Using the arithmetic mean as an estimate of the accuracy for a given noise s.d. appears to result in an underestimate that does not change with increasing noise s.d. (Fig 2D), and hence the mean from the exponential decay of the frequency of Δx was taken to be more representative of the central tendency. To test if motility affected the positional accuracy, we also evaluated the positional accuracy of particles undergoing a random walk (as described in the Materials and Methods section) with a fixed image noise (noise s.d. 30). By increasing the s.d. of the random walk we estimated the effect of increasing velocity on Δx (Fig 3A). The accuracy of positional detection using both the arithmetic and exponential mean error (<Δx >) as before, is less than 1 pixel for the chosen range of velocities of the random walk (Fig 3B). At higher velocities, the tracking errors accumulate, suggesting image noise is the major limiting factor for the positional accuracy of detection, independent of particle motility. Thus, while AMTraK analysis can result in sub-pixel accuracy of position detection, it is essential that the input data have low-noise. We proceeded to test our method on the multiple experimental datasets to examine the utility of this program involving bacterial DNA segregation, microtubule motility and vesicle assembly and transport dynamics.

### Detecting splitting events in bacterial DNA-segregation

**Fig 4:**
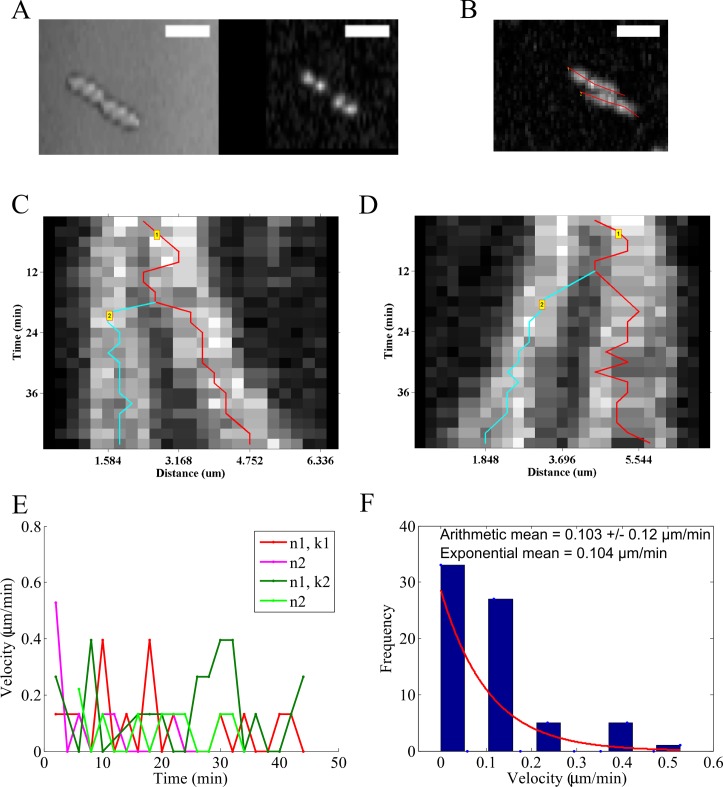
Nucleoid segregation dynamics of *E*. *coli*. (A) Image time-series of *E*. *coli* MG1655 grown on agar pads and imaged in DIC (left) and fluorescence based on HupA-GFP (right) are analyzed using AMTraK. (B) AMTraK generates a maximum intensity projection on the basis of which user-selected lines of interest (red lines) are used by the program to generate kymographs. The kymographs based on (C) LOI 1 (k1) and (D) LOI 2 (k2) were tracked resulting in branched tracks (colored lines). (E) The instantaneous velocities of nucleoids 1 and 2 (n1, n2) from kymographs 1 (k1) and 2 (k2) are plotted as a function of time (colors indicate nucleoids n1, n2 each from the kymographs k1, k2). (F) Mean velocities are estimated using both the arithmetic mean (±s.d.) and v_ex_, the mean of the exponential decay (y = e^-1/vex^) that was fit (red line) to the frequency distribution of instantaneous velocity (bars). Scale bar 4 μm.

A time-series of growing *E*. *coli* is acquired in fluorescence (Movie A in S1 Video) and DIC (Movie B in S1 Video) to follow the nucleoid segregation dynamics of HupA-GFP labeled DNA (Fig 4A). Using the maximum intensity projection produced from AMTraK, the LOIs are chosen (Fig 4B) and used to generate and analyze two kymographs (Fig 4C and 4D). The segregation of the genome is captured by the branched structures of the tracks marked in the kymographs. Additionally we can evaluate both the instantaneous velocity for time-dependence (Fig 4E) and average statistics (Fig 4F). The mean nucleoid transport velocity is 0.103±0.12 μm/min (arithmetic mean ± standard deviation). Based on the form of the frequency distribution of instantaneous velocities, we also fit an exponential decay function to obtain the exponential mean velocity v_ex_ = 0.104 μm/min. These values of nucleoid movement speed from *E*. *coli* MG1655 (wild-type) cells are comparable to a previous report in which nucleoids were tracked in 3D over time [11]. While nucleoids form a diffraction-limited spot in microscopy images, un-branched cytoskeletal filaments form typical 1D structures and dynamics of transport on them and of the filaments themselves, are ideally suited for kymography.

### Microtubule transport: filament edges, centers and time-dependence of velocity

**Fig 5:**
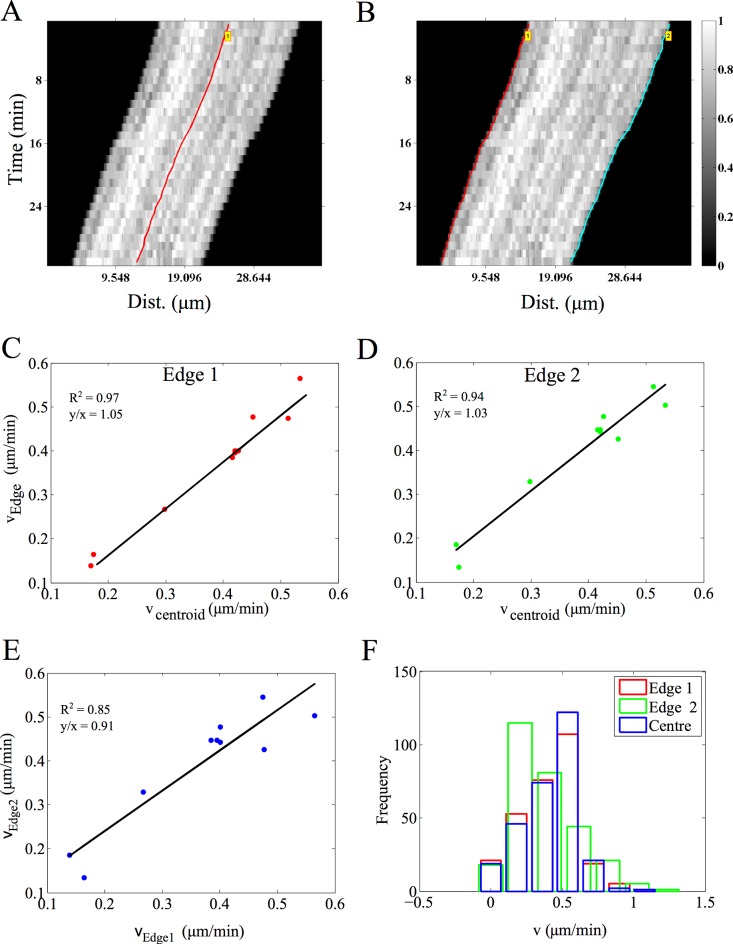
Microtubule (MT) gliding motility on kinesin motors. MTs gliding on kinesin (images acquired every 1 minute for 30 minutes) were analyzed using AMTraK by either detecting **(A)** the centerline (red) or **(B)** the two edges the filament, edge 1 (red) and 2 (cyan). Color bar: gray scale image intensity normalized by the maximal value for the bit-depth. **(C, D)** The velocity estimates from the centroid-based velocity estimates and the two edges and **(E)** the velocity estimated from each edge are correlated. **(F)** The frequency distribution of the instantaneous velocity estimates using the centroid (blue) is compared to edge-based estimates. r^2^: goodness of fit, y/x: slope of the linear fit. Number of filaments analyzed, n = 10.

The transport of microtubule (MT) filaments by surface-immobilized molecular motors in the presence of ATP and buffers is referred to in the literature as ‘gliding assay’ or ‘collective transport assay’. Here, we analyze the gliding motility of MT on kinesin, as described in the methods section, using AMTraK. The analysis of a representative kymograph using either peak- (Fig 5A) or edge-detection (Fig 5B) successful traces the centroids and edges respectively. The mean velocity estimates for collective motor transport show variations between individual filaments. The centroid and edge velocity estimates of multiple MT filaments (n = 10) are strongly correlated as evidenced by the straight line fit with slope ~ 1 (Fig 5C and 5D), as expected. However, the linear correlation of edge-based velocities has a slope of ~0.9 (Fig 5E), suggesting small deviations from the ideal slope, within the range of the average positional detection error (Fig 2C). While typical kymograph analysis of cytoskeletal transport averages the edge information (movement of the tips over time), correlating edge-velocities could potentially be used to estimate small alterations in the filament geometry such as bending and length change. The mean velocity of 0.5 μm/min obtained from our analysis of the assay (Fig 5F) is consistent with previous reports for the same construct [37,38]. While the transport of effectively 1D MT filaments lends itself to kymography, we proceeded to investigate if 2D radial MT structures or asters can also be analyzed by kymography.

### Fusion of MT asters

**Fig 6:**
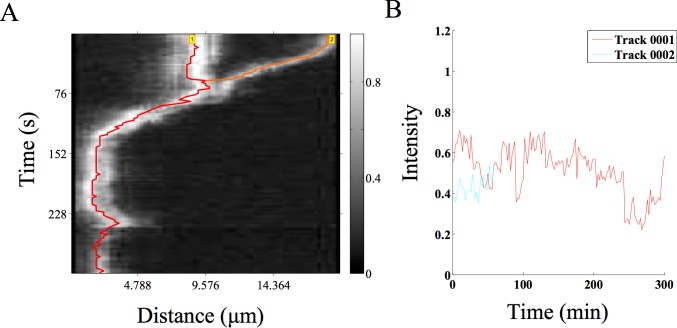
MT aster coalescence. **(A)** A time-series of MT asters undergoing fusion (time-series taken from previous work by Foster et al. [39]) was analyzed using AMTraK. The grey scale bar indicates normalized fluorescence intensity of Alexa-647 labeled tubulin. **(B)** The relative intensity over time of the two coalescing asters is plotted.

In recent experiments by Foster et al. [39] they examined the spontaneous contraction dynamics of radial MT arrays or asters labeled with Alexa647-tagged tubulin, in *Xenopus* egg extracts. We have taken a time-series of such asters from published data (kindly shared by the author Peter J. Foster) and analyzed coalescence events using AMTraK (Fig 6A) The projection of the time-series for selecting the LOI enables us to reduce the complex movements of such 2D structures to a 1D over time process. The movement of the smaller aster as it merges with the larger one is rapid. The fluorescence intensity following the merger fluctuates, but does not increase, which we interpret to mean tubulin density at the center of the new aster does not increase (Fig 6B). While the coalescence appears not to result in a compaction of the aster, it demonstrates the utility of the code for 2D MT array transport. On the other hand, intensity measurements are expected to change during processes such as molecular ‘recruitment’ of sub-cellular structures, so we proceed to test the tool on this process, which had previously been studied using manual kymography.

### Kinetics of clathrin assembly during in vitro vesicle formation

**Fig 7:**
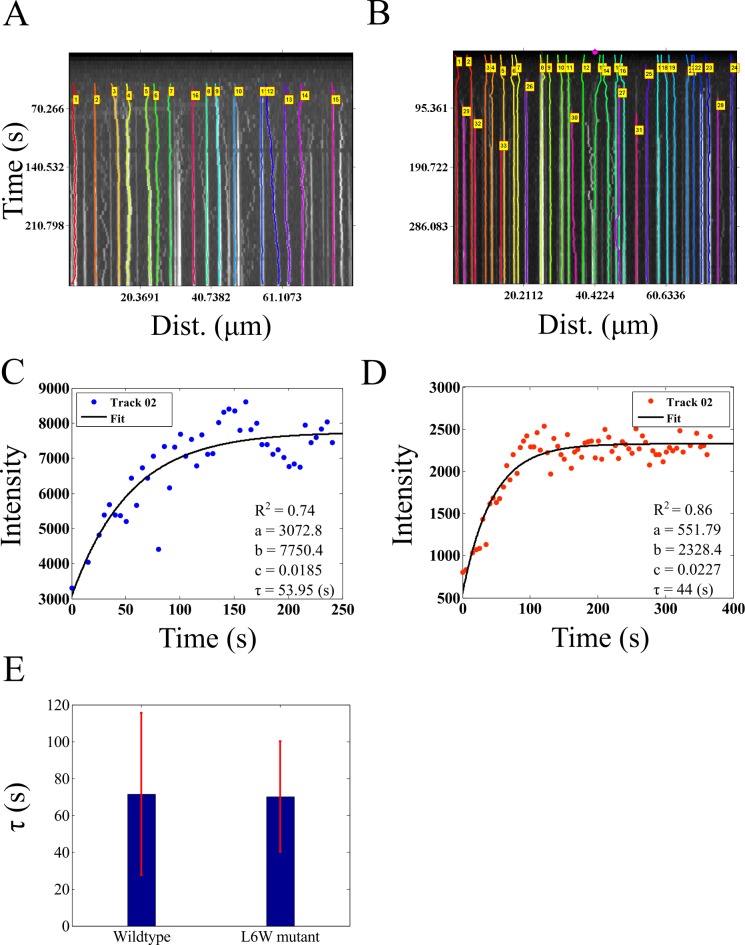
Dynamics of clathrin assembly. **(A, B)** Microscopy time-series taken from Holkar et al. [24] of fluorescently labeled clathrin assembly in the presence of **(A)** wild-type and **(B)** mutant epsin were analyzed using AMTraK. Colored lines in the kymographs indicate detected tracks. **(C, D)** The change in intensity as a function of time based on AMTraK detected tracks from **(C)** clathrin + w.t. epsin and **(D)** compared to clathrin + (L6W) mutant epsin. The intensity kinetics plots are fit to a single-phase exponential function, y = a+(b-a)*(1-e^-c*t^) to obtain the time constant of assembly τ = 1/c (red). R^2^: goodness of fit. **(D)** The mean values (error bar represents s.d.) of the time constant of assembly of clathrin (τ) in the presence of wild-type and mutant epsin are compared.

We proceed to quantify the assembly kinetics of clathrin on membranes from an *in vitro* reconstitution assay of clathrin assembly on vesicle precursors reported previously by Holkar et al. [24]. This process has been analyzed using kymography due to its effectively 1D spatial extent and the multiple simultaneous events of assembly. The published time-series of fluorescently labeled clathrin assembly kinetics in the presence of wild-type epsin (supplementary movie 3 in [24]) and L6W mutant epsin (supplementary movie 5 in [24]) in the form of 16 bit TIF images were provided by the authors (Sachin Holkar, personal communication). AMTraK was used to analyze this data without any pre-processing, resulting in tracked kymographs of assembly kinetics with wild-type (Fig 7A) and mutant epsin (Fig 7B). The software outputs a text-file of grey-value intensities normalized by the bit-depth (maximum normalized, between 0–1) (S4 Data), which when multiplied by the bit-depth of the input images, produced intensity profiles of clathrin assembly in grey-values with time in the presence of wild-type (Fig 7C, S3A Fig) and mutant epsin (Fig 7D, S3B Fig). These intensity profiles were fit to a single phase exponential function y = a+(b-a)*(1-e^-c*t^), where y is the intensity which increases with time t, and depends on three fit parameters, a, b and c, the same function as used by Holkar et al. [24]. A large proportion of the assembly events were successfully tracked and most showed saturation kinetics that were fit by curves with R^2^>0.7 (S3 Fig). While the parameters *a* and *b* are scaling factors, *c* determines the characteristic clathrin polymerization time, τ = 1/c. In our analysis the clathrin assembly time in presence of wild-type epsin is <τ> = 71.49±44.09 s while with mutant epsin <τ> = 70.16±29.89 s. In our estimate of the mutant assembly time is indistinguishable from wild-type, consistent with the previous report, which used manual quantification of the kymograph [24]. We proceed to examine if our tool, which appears to work successfully on *in vitro* data with low background noise, can also be used for the quantification of *in vivo* dynamics inside the crowded environment of an intact cell.

### Axonal vesicle transport: Characterizing directional switching

**Fig 8:**
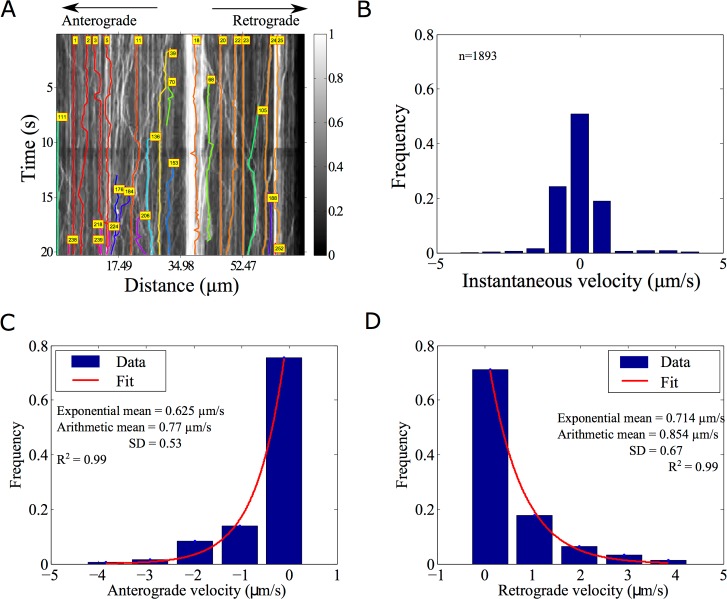
Analysis of synaptic vesicle transport. **(A)** GFP-Rab3 tagged vesicles from posterior touch cell neurons in *C*. *elegans* (experimental data from taken from supporting movie S1 Movie from [28]) were analyzed using AMTraK. Colored lines with index numbers indicate tracks. **(B)** The frequency distribution of instantaneous velocities of the vesicles (n = 1592) is plotted using AMTraK (mean: 0.49 μm/s, s.d. 0.88). **(C, D)** The frequency distribution of non-zero velocities are fit with an exponential decay function y = A*e^-x/m^ (red line), where A: scaling factor and *m*: mean. **(C)** The mean anterograde velocity from the fit is 0.625 μm/s with arithmetic mean 0.77±0.53 μm/s (n = 425) and **(D)** the mean retrograde velocity from the fit is 0.714 μm/s with arithmetic mean 0.854±0.67 μm/s (n = 540). Arithmetic means are reported ± standard deviation (s.d.). R^2^ indicates the goodness of the fit.

Synaptic vesicles in *Caenorhabditis elegans* mechanoreceptor neurons labeled with GFP-Rab3 have been recently studied by Mondal et al. in a whole-animal microfluidics device, providing retrograde and anterograde vesicle transport statistics [28]. Such *in vivo* data is complex, involves multiple crossovers and has many objects close to each other. AMTraK based analysis of the published data could detect up to 17 different tracks (Fig 8A). Vesicles that were not detected have typically low intensity or were out of focus and were not segmented. The spread of the distribution of instantaneous velocities (left-ward: negative, anterograde; right-ward: positive, retrograde, non-motile: paused) shows that the GFP-Rab3 vesicles are equally likely to be anterograde and retrograde in their transport (Fig 8B). Based on the shape of the frequency distribution of the non-zero velocities in anterograde (Fig 8C) and retrograde (Fig 8D) directions, an exponential decay fit to the frequency distribution was used to estimate mean velocities (goodness of fit, R^2^ = 0.99). To enable comparison with the arithmetic means reported in literature [28], we also estimate the average. The mean velocity from the exponential fits of anterograde transport is 0.625 μm/s (n = 425, arithmetic mean±s.d.: 0.77±0.53 μm/s) while the mean retrograde velocity is 0.714 μm/s (n = 540, arithmetic mean±s.d.: 0.854±0.67 μm/s). In this case, both means are comparable since only non-zero values were the analyzed. Velocities in both directions are of comparable order of magnitude to the published values obtained by manual detection [28], but 1.5-fold lower, due to a (non-zero) threshold velocity used by the authors to define pauses (as personally communicated by the author, Sudip Mondal). Thus, AMTraK can be reliably used to quantify transport and assembly dynamics from both *in vitro* and *in vivo* fluorescence microscopy data, as seen from the quantification, which is consistent with literature.

## Discussion

In this report, we have described a novel tool for automatic detection and quantification of kymographs from fluorescence microscopy time-series. Using simulations we have demonstrated sub-pixel position detection accuracy of our proposed method, in conditions of low Gaussian noise. The program quantifies position, motility, and brightness intensity of fluorescence signal and fusion/splitting events. The utility of the code is tested on *in vitro* and *in vivo* fluorescence time-series ranging from *in vitro* assays of MT gliding assays with kinesin, coalescence dynamics of MT-asters, clathrin assembly kinetics on lipid tethers to *in vivo* axonal synaptic vesicle transport. The measures of average transport and kinetics of these diverse data types are consistent with published data and provides opportunities for improved statistics of individual events from a dynamic time-series, which were not as easily accessible with current methods.

Manual quantification of kymographs [2] depends typically on reliable edge detection. As a result, quantification varies between individuals and requires prior information or experience [40]. Yet, manual kymography is widely reported in cell-biological literature for the analysis of dynamic processes, possibly due to the heterogeneity of the data types and the absence of a single standard method or even criterion, which to make the process less interactive. While developing AMTraK, we tested global (whole-image) methods of edge-segmentation (contour-, watershed- and gradient-based), but found them to be inadequate for the task. Possible reasons include the time-dependent brightness and contrast changes of the sample resulting from either bleaching or intrinsic dynamics. We find that for some applications such as vesicle transport and protein recruitment, the detecting and tracking peaks is ideal, while for microtubule gliding assays edge detection is better. As a result our code allows the user to choose amongst three different methods of segmentation based on the nature of their data (a) peak detection by *findpeaks* and (b) watershed and (c) edge detection using the Canny edge detector.

Typical problems in peak or edge detection arise when the data has poor signal to noise. This is also seen in our error analysis with increasing noise amplitude (Fig 2D). One solution is to background subtract the image, which can be easily done in multiple tools. The occasional loss of some particles in a time-series such as synaptic vesicles (Fig 8A), despite being visible to the eye, results from a failure in detection or a `pruning’ step used to remove spurious and redundant tracks. Such pruning however was found to be necessary to ensure robustness of the code for handling multiple data types and is simple to trouble-shoot due to the limited number of adjustable parameters. While intensity matching did not improve the percentage vesicles tracked, in future additional features like those used in pattern-matching for tracking [41] could be used further improve the detection percentages. Our test with increasing Gaussian random image noise (Fig 2) also suggests that increases of fluorescently tagged proteins (for instance due to expression level increases *in vivo*), could result in reduced spatial contrast. Such data would then be difficult to automatically quantify using AMTraK. The data would require pre-processing with something similar to an anisotropic diffusion filter [42] to preserve edge information but reduce non-specific signal. In future, multiple data pre-processing routines could be implemented in a separate module, to add to the functionality of the program.

Our quantification of the frequency distribution of synaptic vesicle transport in anterograde and retrograde directions (Fig 8C and 8D) suggests the instantaneous velocities are exponentially distributed. While the arithmetic mean suffices for comparison with experimental reports [28], the quantification of the precise nature of the distribution of velocities could be used as a test of theoretical models. Such a comparison has been made in previous work on synaptic vesicle precursor trafficking [43]. Such models are relevant for both neurophysiology as well as understanding of collective effects in molecular-motor driven vesicle transport *in vivo* [44,45].

The collective motor velocity of human kinesin driven gliding of MTs has been well characterized in previous work [12,46,47]. Many of these studies have shown that the MT length and kinesin density do not affect the mean speed. However, the time-series of individual filaments show small time-dependent variations (Fig 4A and 4B), possibly a result of the local inhomogeneity of motor distributions. This information could be of some use when mixed-motor populations are used [48]. Recent studies of filament motility have used a filament-tracking approach based on a MATLAB program FIESTA [49], with a positional accuracy of 30 nm. We find the distribution of time-averaged velocity of gliding calculated using AMTraK match closely the distribution obtained from analysis using FIESTA (S2 Fig). This suggests that while complex transport dynamics in 2D are indeed better analyzed using tracking tools, for those data sets that are amenable to kymography analysis, AMTraK results are comparable to those obtained from tracking tools with sub-pixel accuracy.

While the dynamics of multiple particles can be simultaneously quantified using AMTraK, the selection of LOIs remains manual. However, once an LOI has been selected, the program can also be used in the “From file” mode to apply a pre-existing LOI to quantify kymographs in other channels (e.g.: bright field, fluorescence) and other fields of view with similar sample geometries. Potentially, LOIs could be generated independent of AMTraK too, provided they are compatible with the input format. The multiple bright-field and fluorescence correlative analysis tools for bacterial image analysis [10,33,50,51] are an example in case. More recent developments in image-analysis software to systematically extract data from microfluidics experiments automatically output channel information [52], which could also form the basis for the LOIs for multiple fields of view. These approaches could in future further increase the throughput our analysis tool.

Multiple software tools for kymography have been described in the recent past in literature and their features are summarized in Table 1 for comparison. While most tools including this one, require user inputs for the process of kymograph generation, only AMTraK and Kymograph Direct [53] automates the detection and connection. However, certain features of AMTraK make it unique, being absent in other comparable tools, such as automated branch-point detection, an integrated quantification module and sub-pixel positional accuracy accessible with an easy to use GUI front-end. In addition, since the code is open source and written in MATLAB, it is more likely to be used in an existing microscopy analysis workflow, due to the increasing spread of MATLAB as a data analysis platform in quantitative cell-biology research [54,55]. Thus, AMTraK could serve as a tool for the rapid quantification of image time-series of transport and assembly kinetics from microscopy. This has become particularly relevant in the context of high-content screening [56], where the spatial interaction patterns are becoming just as important as bulk kinetics measured in traditional high throughput screens.

**Table 1:** A comparison of features in kymography tools described in literature and commonly in use for cellular and sub-cellular scale images.

Feature / Tool	AMTraK	Multi-kymograph	Makekymograph	Icy- Kymograph Tracker	Kymomaker	Points from Kymograph	Kymograph mt2	KymographClear and KymographDirect
LOI selection	Manual	Manual	Semi-automated	Manual	Manual	No	Manual	Manual
Multiple LOIs	Yes	Yes	Yes	Yes	Yes	No	No	No
Automated track detection	Yes	No	No	Semi-automated	Yes	Semi-automated	No	Yes
Quantification	Yes	Separate	No	Separate	No	XY-coordinates	No	Separate
No. of adjustable parameters	8	1	1	7	13	-	-	-
Split and merge detection	Automatic	No	No	No	No	No	No	Manual
Open source	Yes	Yes	Yes	Yes	No	Yes	Yes	Yes
Programming language	MATLAB	ImageJ macro	Java (ImageJ plugin)	Plugin for Icy	-	Java (ImageJ plugin)	Java (ImageJ plugin)	ImageJ macro and LabView
Reference	This report	[57]	[58]	[5]	[4]	[59]	[60]	[53]

We have developed an automated tool for the quantification of kymographs. Our approach detects peak and edge information and utilizes a distance minimization approach to link them. We demonstrate the wide utility of our tool by quantifying microtubule transport dynamics, clathrin polymerization kinetics and vesicle transport. Combined with a user-friendly interface, objective detection criteria and open source code, we believe AMTraK can be used to extract more and reproducible statistics from microscopy of sub-cellular dynamics.

## Supporting Information

S1 Data The LOI coordinates generated are stored in the file “LOIselection.txt” when the user chooses the “Interactive” mode of LOI selection at the stage of generating a kymograph. This provides the 2D image coordinates (X and Y) in pixel units, as indicated by the columns labels.
(TXT)

S2 Data The average statistics for all trajectories are stored in a file “USER_TrackStats.txt”. It reports in a column-wise manner the track number, time over which it is tracked (in user-provided units), speed, net-velocity (displacement/time), tortuosity (displacement/path-length), average of instantaneous velocity and the standard deviation of the average instantaneous velocity. All column headers are labeled for clarity.
(TXT)

S3 Data The instantaneous (time-dependent) statistics of each track are stored in “USER_InstStats.txt” with track number, time interval to the previous frame in units provided by the user, displacement magnitude, positive/negative displacement (leftwards: negative, rightwards: positive), instantaneous velocity (displacement/time interval), signed-velocity (leftwards: negative, rightwards: positive), and cumulative time (adding up time intervals in units provided by the user). All column headers are labeled for clarity.
(TXT)

S4 Data The file “Tracklist.txt” stores the time-dependent intensity statistics of each track. This provides the track number, position in distance from the origin (upper-left corner) in pixels, time-frame (frame number), normalized grey-value intensity (divided by the bit-depth of the image) and normalized time-frame (setting the first time-frame to 0). All column headers are labeled for clarity.
(TXT)

S5 Data The branch-points detected by the code are stored in a file “Branchpoints.txt” which is generated when the user chooses to detect “Splitting events” (check-box) with an appropriate parameter choice. It contains the track-number that splits off from or joins another track, the 1D distance (from the origin at the left edge) and it’s time point both in terms of user-provided units. The column headers describe the variables.
(TXT)

S6 Data User provided values are stored in “All_Parameters.txt”. This includes the name and path of the input TIF image time-series, scaling factors (distance, time) and parameters for the detection, tracking and splitting-events.
(TXT)

S1 Fig The simulated image. **(A)** The simulated bead image used to estimate the accuracy of the code. A profile through the image (yellow line) is used to generate **(B)** an intensity profile through the three beads.
(PDF)

S2 Fig Comparing kymography to filament tracking. The frequency distribution of instantaneous velocities obtained after analyzing time-series of MTs gliding on kinesin using AMTraK (red bars) and the high-precision filament-tracking tool, FIESTA (blue bars) are plotted.
(PDF)

S3 Fig Kinetics of clathrin endocytosis. The fluorescence intensity in grey values (colored circles) as a function of time in seconds estimated from multiple detected tracks after AMTraK analysis (Fig 7A and 7B) of clathrin assembly kinetics in the presence of **(A)** wild-type and **(B)** mutant (L6W) epsin (based on data from Holkar et al. [24]). A single-phase exponential function (the same as in Fig 7C and 7D) is used to fit the data (black line) and the parameters are listed for each fit, with τ indicating the time-constant of assembly in seconds.
(PDF)

S1 Video Time-series of division and genome-segregation in *E*. *coli* MG1655 is followed **(A)** in fluorescence with nucleoids labeled by HupA-GFP (grey) and **(B)** DIC is used to follow cell morphology. Scale bar: 4 μm. Time indicated in minutes.
(ZIP)
