# Supplementary figures and images for: Automated Multi-Peak Tracking Kymography (AMTraK): A Tool to Quantify Sub-Cellular Dynamics with Sub-Pixel Accuracy
Source: PLoS One. 2016 Dec 19;11(12):e0167620. doi: 10.1371/journal.pone.0167620 (PMC5167257; doi:10.1371/journal.pone.0167620)

A

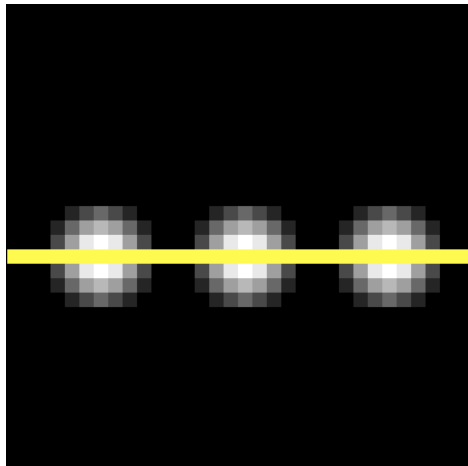

B

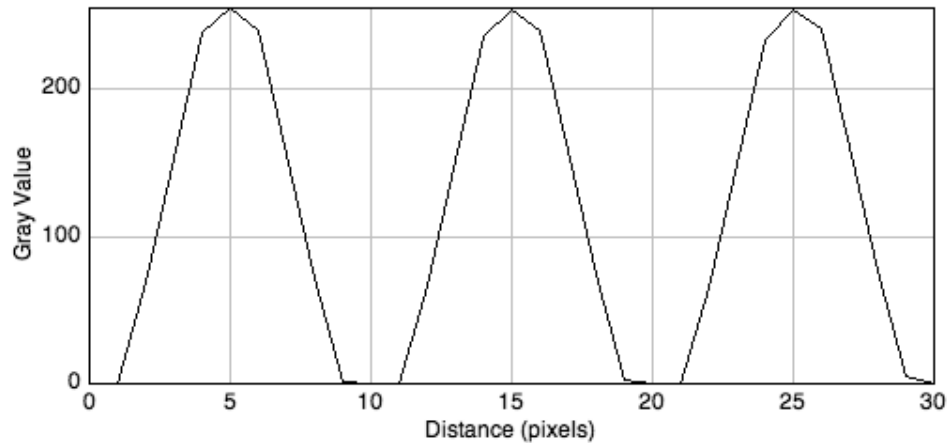

Supplement: S1 Fig — (A) The simulated bead image used to estimate the accuracy of the code. A profile through the image (yellow line) is used to generate (B) an intensity profile through the three beads. (PDF) [file pone.0167620.s007.pdf]

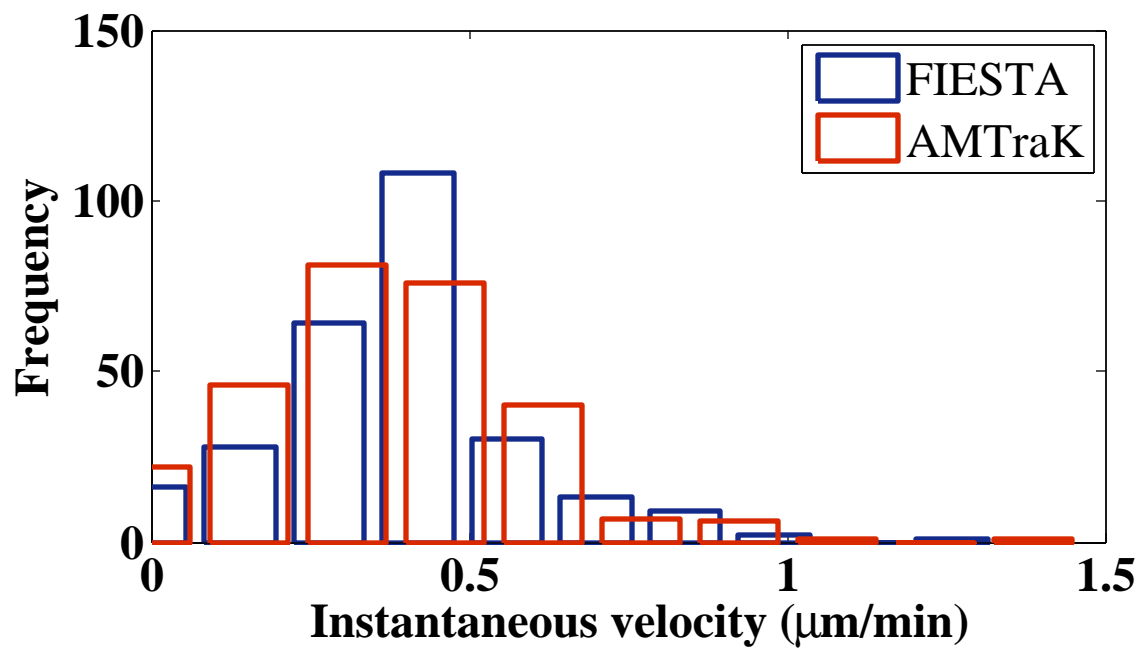

Supplement: S2 Fig — The frequency distribution of instantaneous velocities obtained after analyzing time-series of MTs gliding on kinesin using AMTraK (red bars) and the high-precision filament-tracking tool, FIESTA (blue bars) are plotted. (PDF) [file pone.0167620.s008.pdf]

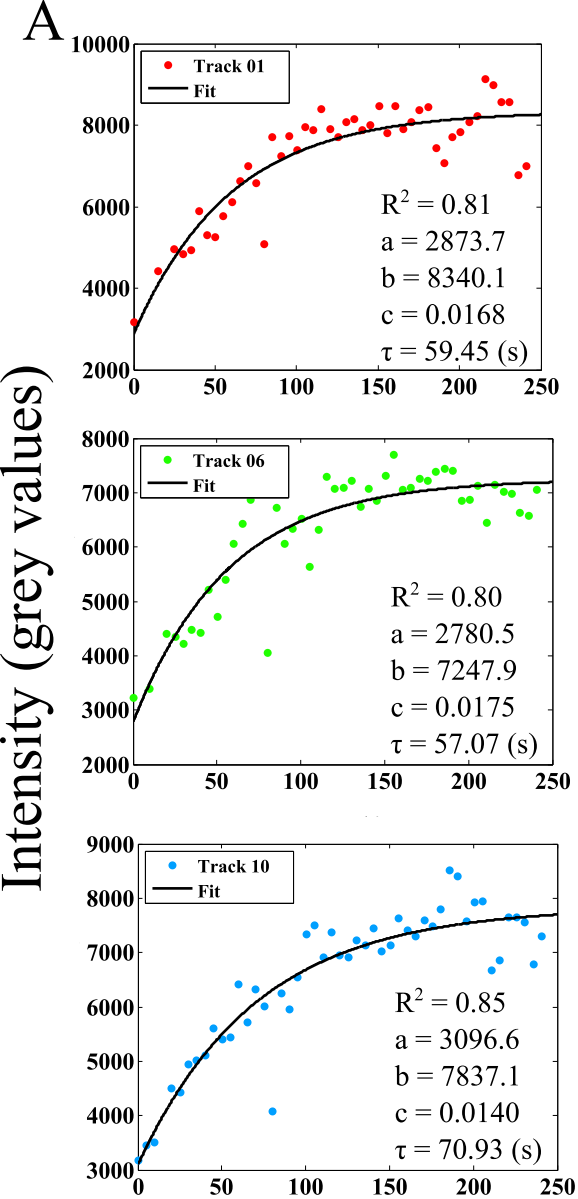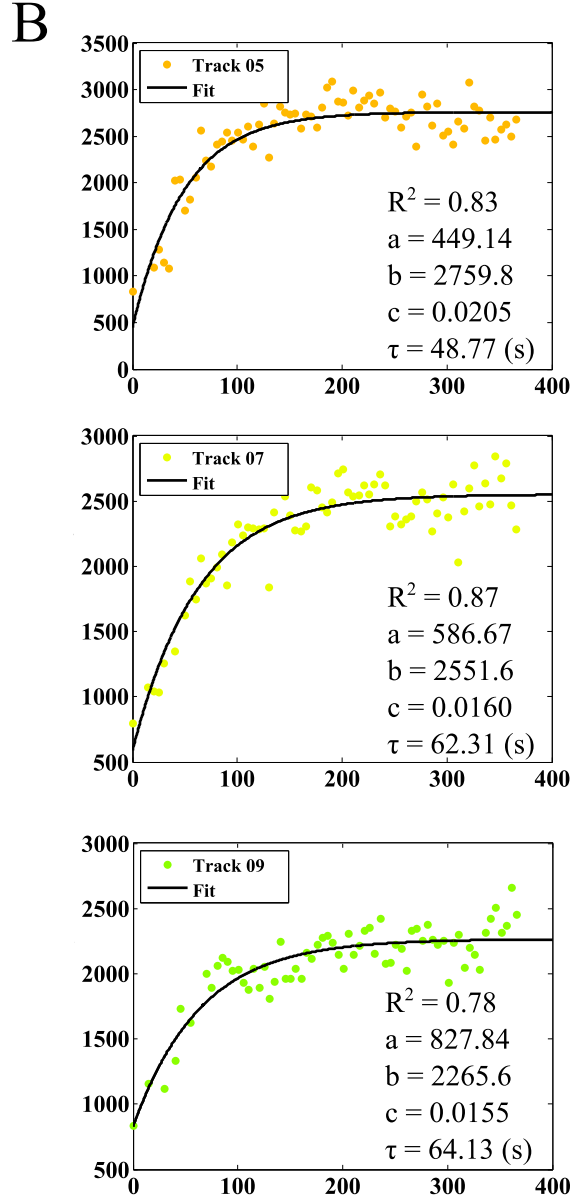

Time (s)

Supplement: S3 Fig — The fluorescence intensity in grey values (colored circles) as a function of time in seconds estimated from multiple detected tracks after AMTraK analysis (Fig 7A and 7B) of clathrin assembly kinetics in the presence of (A) wild-type and (B) mutant (L6W) epsin (based on data from Holkar et al. [24]). A single-phase exponential function (the same as in Fig 7C and 7D) is used to fit the data (black line) and the parameters are listed for each fit, with τ indicating the time-constant of assembly in seconds. (PDF) [file pone.0167620.s009.pdf]
